# Silicea

**Published:** 1898-07

**Authors:** 


### HOMŒOPATHIC PHYSICIAN,

A MONTHLY JOURNAL OF

HOMŒOPATHIC MATERIA MEDICA AND CLINICAL MEDICINE.

"If our school ever gives up the strict inductive method of Hahnemann, we are lost, and deserve only to be mentioned as a caricature in the history of medicine."—CONSTANTINE HERING.

Vol. XVIII. JULY, 1898. No. 7.

### EDITORIAL.

SILICEA has amelioration from standing and from rapid motion like dancing. Sulphur has aggravation from standing.

Silicea is an antidote to large doses of Mercury and Sulphur. Fluoric-acid and Hepar are antidotes to Silicea.

The following are Dr. Guernsey's key-notes to Silicea: Acrid leucorrhœa. Itching of the vulva. Constipation; the stool when partially evacuated slips back again. Tumor of the vulva with suppuration, which is increased by walking. The affected parts are very sensitive to touch. All symptoms are worse at the new moon. Violent burning and soreness of the genitals, with eruption on the inner surfaces of the thighs. More or less itching of the vagina, with fever all night. Emaciation and gradual failing of health. Labor-like pains in the vagina, with sensitiveness to touch. Vaginal fistula with great tenderness, and of the parts around it. Scrofulous diathesis. A pressing-down feeling in the vagina. Prolapsus uteri in consequence of myelitis. Very difficult stools. Painful smarting leucorrhœa, after taking acids.

Leucorrhœa during urination. Milky leucorrhœa in paroxysms, preceded by cutting around the umbilicus. Discharge of blood between the menstrual periods.

Increased menses with repeated paroxysms of icy coldness over the whole body. Attacks of melancholy. Anguish in the pit of the stomach. She wishes to drown herself. Fœtid, brownish, purulent, ichorous leucorrhœa. Constipation occurring immediately before and during the menses. Momentary attacks of sudden blindness or of obscured vision. Discharge of a quantity of colorless or white water from the uterus instead of the menses. Stool of hard lumps remaining long in rectum from apparent want of power to expel it. Burning soreness of the vulva. Eruption on inner sides of thighs during the menses. The menses have a very strong smell.

Metrorrhagia with terribly offensive sweating of the feet. Hungry, but cannot swallow the food, it seems so nauseous. Nausea with violent palpitation of the heart. Nausea after any exercise that raises the temperature of the body. In the morning taste of blood in the mouth. Sore, sticking, shooting pains at every effort at stool. Loss of hearing, partially relieved by blowing the nose. She occupies herself with pins; she hunts them and counts them; she is worse during the increase of the moon. Pure blood is passed from the uterus every time the infant nurses. The nipple ulcerates very easily, and it is very sore and tender. Fistulous ulcer of the breast, the discharge being thin and watery, or thick and offensive. One lobe of the mammary gland after another seems to ulcerate and discharge into one common ulcer, often with great pain. There may be several orifices, one for each lobe. Bronchitis in children that have large bellies. Perspiration about the head. Suppuration of the glands. Frequent attacks of colic, relieved by discharges of offensive flatus. Spasms of children which return at change of moon. Pot-bellied children.

